# Sera from severe trauma patients with pneumonia and without infectious complications have differential effects on neutrophil biology

**DOI:** 10.1186/s12890-016-0329-7

**Published:** 2016-12-01

**Authors:** B. Relja, R. Taraki, M. P. J. Teuben, K. Mörs, N. Wagner, S. Wutzler, F. Hildebrand, M. Perl, I. Marzi

**Affiliations:** 1 Department of Trauma, Hand and Reconstructive Surgery, Goethe University, Theodor-Stern Kai 7, 60590 Frankfurt, Germany; 2 Department of Orthopaedic Trauma, University Clinic RWTH Aachen, Pauwelsstraße 30, 52074 Aachen, Germany; 3 Department of Trauma Surgery, Trauma Center Murnau, Murnau, Germany

**Keywords:** Trauma, Pneumonia, Neutrophils, CD31, CD62L, Migration, Oxidative burst

## Abstract

**Background:**

Major trauma patients (TP) developing imbalanced immune response are at high risk for infectious post-injury complications including pneumonia. Neutrophils play a central role in the host defense against bacteria and thereby pathogenesis of infections. While there are numerous studies about neutrophil function after trauma, data about their biology in patients who suffer from pneumonia following trauma are sparse. Here, we studied the effect of serum isolated from patients who do and do not develop infection (inf.) on the biology of neutrophils from healthy volunteers.

**Methods:**

Sera samples from eighteen TP with an injury severity score above 16 were obtained. Nine patients were grouped to *no inf.* group (TP without pneumonia), and nine to *inf*. group (TP with pneumonia). Samples were obtained at admission to emergency department (ED), a day prior pneumonia diagnosis (*1 d prior inf*) or at the day of diagnosis (*1 d prior inf*). Samples from the equal post-injury days in the corresponding *no inf*. group were used. Neutrophils from nine healthy volunteers were isolated. Effects for sera isolated from infected and non-infected patients on neutrophil biology were analyzed. Migratory capacity of neutrophils towards TP’s serum, their CD11b and CD62L membrane receptor expression and oxidative burst activity after stimulation with TP’s serum were determined and compared between groups.

**Results:**

Migratory capacity of neutrophils was significantly increased after trauma and persisted during the study period. CD11b expression in all groups was significantly increased. CD62L expression decreased generally in samples from ED and recovered later to baseline. Stratifying *no inf*. and *inf*. groups showed significantly decreased migratory capacity, increased CD11b and significantly decreased CD62L expression in the *no inf*. group. These differences persisted during the complete observational period. ROS production was strongly reduced in the *no inf*. group compared to the *inf*. group at later experimental time points.

**Conclusions:**

This data indicate that patients at risk for pneumonia development have differentially and early activated neutrophils following trauma compared to patients who are not at risk for post-injury complication. Studies about the differential biology of neutrophils and their immediately after trauma modified activity depending on the post-injury clinical course are warranted, and may deliver predictive or even therapeutic strategies to control inflammation.

## Background

Trauma is a leading cause of death worldwide [1, 2]. Despite continuous advances in trauma care systems and emergency medical services, severely injured trauma patients (TP) who survive hospital admission are highly susceptible to secondary complications, such as nosocomial infections and/or multiple organ dysfunction syndrome (MODS) [3–5]. In response to traumatic injury and the associated release of damage-associated molecular patterns (DAMPs), the body’s immunological and inflammatory processes at cellular and humoral levels become activated in order to neutralize non-infectious tissue insult, bacterial infections, and initiate tissue repair mechanisms [6, 7]. This post-injury process termed as the systemic inflammatory response syndrome (SIRS) is simultaneously counterbalanced by a compensatory anti-inflammatory response syndrome (CARS) resulting in a mixed antagonist response syndrome (MARS).

Neutrophils, critical inflammatory cells playing a key role in protection against bacterial infections, are believed to be central in the pathogenesis of infectious post-injury complications. Due to their numerous microbicidal defensive strategies including production of reactive oxygen species (ROS), neutrophils constitute the first line of defense against rapidly dividing bacteria, fungi and yeast [8]. Following trauma, several studies have reported altered functions of neutrophils, which are believed to promote secondary post-injury complications [9–11]. Pulmonary complications such as e.g., acute respiratory distress syndrome are characterized by increased numbers of activated neutrophils in lungs as well as their increased respiratory burst activity [12]. Recently, it has been reported that the neutrophil oxidative burst as response to orthopaedic trauma surgery was associated with infectious post-injury complications pneumonia and sepsis [13]. Not only the increase in their oxidative burst activity but also their increased migratory capacity across damaged endothelium and sequestration in bystander organs after trauma, characterize their importance in post-injury inflammation and secondary infections. Both, migration and transmigration of neutrophils from blood into tissues include tightly controlled interactions between their surface adhesion molecules and the corresponding ligands on endothelial cells. Initial rolling and capturing process is mediated by selectins including CD62L (L-selectin). Following different trauma mechanisms, including penetrative as well as blunt trauma and traumatic brain injury, L-selectin expression on circulating neutrophils has been reported to be significantly reduced [14, 15]. On the other hand, the firm adhesion and transmigration requires integrins as e.g., CD11b [16]. As a marker of neutrophil activation after trauma elevated surface expression of CD11b that forms part of the heterodimeric integrin Mac-1 (macrophage associated antigen-1) was reported after TBI and thermal injury [14, 17]. Others reported that CD11b although initially unchanged, became down-regulated on day 3 following major trauma [18]. However, in their recent review article, Hazeldine et al. postulate that combining the data for L-selectin and increased CD11b expression suggests a systemic activation of circulating neutrophils after trauma [12]. Interestingly, although, there are numerous reports about neutrophil function after trauma, the human studies about the predictive potential of their modulated biology for the outcome after trauma are sparse and focus rather on cell-free DNA [19, 20]. Although our understanding of the neutrophil function after trauma is increasing, the understanding of their functional modulations in patients who are susceptible to infection following trauma requires further studies.

## Methods

### Patients

In this experimental trial 18 severely injured trauma patients with a history of acute blunt or penetrating trauma and an injury severity score (ISS) ≥ 16 were enrolled. Exclusion criteria were younger 18 or older 80 years of age, severe burn injury, acute myocardial stroke, cancer or chemotherapy, immunosuppressive drug therapy, HIV, infectious Hepatitis, acute CMV infection and/or thromboembolic events. Vital signs were measured and the ISS was calculated according to the abbreviated injury scale (AIS, 2008) upon arrival at the emergency department (ED) [21–23]. A subgroup analysis after frequency-matching of patients according to the ISS (± 6 points) and age was performed. The presence of post-traumatic pneumonia development was defined by radiologic, clinical and bacteriologic findings with the presence of new pulmonary infiltrates on chest X-ray and at least one of the following criteria: positive blood culture, bronchial alveolar lavage and/or sputum culture [24].

### Blood sampling

Blood samples were withdrawn in S-Manovette® Z-Gel tubes (Sarstedt, Nürmbrecht, Germany) directly after admission to the ED and daily until day 10 after trauma. Blood was centrifuged at 2000 x g for 15 min at 4 °C. The supernatant was stored at -80 °C until sample use for further stimulation experiments. The subsequent blood samples taken daily from TP were obtained between 7 and 11 a.m. Serum samples were used for stimulation of isolated neutrophils from healthy volunteers. The samples that were obtained from TP at admission to the emergency department (ED), one day prior diagnosis of pneumonia (inf., 1 d prior inf) or at the same day in the corresponding group of patients without pneumonia (no inf.) (1 d prior inf), or at the day of pneumonia diagnosis (inf., d of inf) or at the same day in the corresponding group of patients without pneumonia (no inf.) (d of inf) were used for experiments.

Blood samples from healthy volunteers were withdrawn in heparinized tubes (Sarstedt, Nürmbrecht, Germany) and kept at room temperature until isolation of neutrophil granulocytes. The blood samples taken from HV were obtained between 7 and 11 a.m.

### Isolation of neutrophils

The isolation of blood neutrophils was performed by density-gradient centrifugation (Polymorphprep, Axis-Shield, Oslo, Norway) according to manufacturer’s instructions. In short, 4 ml of Polymorphprep (density: 1.113 ± 0.001g/ml) were covered carefully with 4 ml heparinized whole blood from HV. After sample centrifugation for 30 min as suggested by manufacturer, the PMN cell fraction was removed and transferred to another tube for another washing procedure with phosphate buffered saline w/o Ca^2+^ and Mg^2+^ (Invitrogen). Subsequently, supernatant was removed and PMN were cultured in RPMI 1640 (Seromed, Berlin, Germany; polypropylene tube, BD Bioscience, Franklin Lakes, NJ, USA) supplemented with 10% heat-inactivated fetal calf serum (FCS), 100 IU/mL penicillin and 100 μg/mL streptomycin (Gibco, Karlsruhe, Germany) and 20 mM HEPES buffer (Sigma, Deisenhofen, Germany) medium and their number as well as their viability was determined by the trypan blue exclusion assay. For the analysis of the effects of sera obtained from TP, PMN were cultured in media containing 20% of TP serum. Only cell cultures with a purity of >95% were utilized for experimental use.

### Migration assay

Serum induced cell migration was examined using 24-well Transwell chambers (Corning, New York, USA) with 3-μm pores. Before the evaluation of the migratory rate of neutrophils, cell number was calculated to 1 × 10^6^ vital cells/ml. Subsequently, 100 μl of the cell suspension were placed in the upper chamber. The lower chamber contained 500 μl RPMI 1640 with supplements and 20% serum obtained from trauma patients. After incubation for 3 h at 37 °C and 5% CO_2_, the Transwell membrane was removed and cells migrating to the lower chamber were collected and counted after their staining with Türks-solution (1 : 1, Merck). The number of cells migrating into the lower compartment was quantified. The migratory capacity of control (ctrl) cells that were incubated without serum in the lower compartment was set as 100%. Graphical results are shown as % migration compared to the 100% ctrl.

### Measurement of cell surface receptor expression by flow cytometry

Isolated neutrophils (2 × 10^6^ cells/ml) were transferred in 250 μl RPMI 1640 (polypropylene tube, BD Bioscience, Franklin Lakes, NY, USA) with supplements as described above including 20% serum from trauma patients. Control samples were incubated in medium containing supplements without the serum from trauma patients. The samples were incubated at 37 C° and 5% CO_2_. Two hours later, the samples were centrifuged at 2100 g for 15 min, the supernatant was removed and the cells were transferred into polystyrene FACS tubes (BD Pharmingen™, Heidelberg, Germany) for flow cytometry staining. The neutrophils were incubated with mouse anti-human CD62L APC (Clone DREG-65) and mouse anti-human CD11b/Mac-1 PE (Clone ICRF44, all BD Pharmingen, Heidelberg, Germany) antibodies. After 30 min at room temperature, the cells were washed with 4 ml phosphate buffered saline supplemented with 0.5% bovine serum albumin (FACS buffer) and centrifuged at 400 g for 5 min. The supernatants were removed, the cells diluted in 300 μl FACS buffer and subjected to flow cytometry using BD FACS Canto 2*™* and FACD DIVA™ software (BD). The neutrophils were gated by the corresponding forward- and side scatter scan. Mean fluorescence unit (MFU) was detected. From each sample a minimum of 20.000 cells was measured. The number of totally gated cells for each sample was calculated as absolute cell number in percentage relative to the ratio of the indicated cell populations in representative figures.

### Oxidative burst

Isolated neutrophils (2 × 10^6^ cells/ml) were cultured as described above with/without 20% serum from trauma patients in culture media. Control samples were incubated in medium without serum from patients. The samples were incubated at 37 °C and 5% CO_2_ for 2 h later centrifuged at 2100 g for 15 min, resuspended in culture medium (1 ml) with supplements and 100 μl of the cell suspension were transferred into polystyrene FACS tubes. Twenty μl CM-H2DCFDA (CM-H_2_DCFDA, General Oxidative Stress Indicator Kit, Invitrogen, Darmstadt, Germany) were added to each sample as suggested by the manufacturer. Subsequently, the samples were incubated for 30 min at 37 °C and 5% CO_2_. Thereafter, 400 μl of cell culture medium with supplements (as described above) were added to each sample. After 60 min at at 37 °C and 5% CO_2_, the cells were washed with 4 ml FACS buffer and centrifuged at 400 g for 5 min. The supernatants were removed, the cells diluted in 200 μl FACS buffer and subjected to flow cytometry using BD FACS Canto 2*™* and FACD DIVA™ software (BD). The neutrophils were gated by the corresponding forward- and side scatter scan. From each sample a minimum of 20.000 cells was measured. The amount of positive cells for oxidative stress was calculated relative to the whole neutrophil population of unstained cells as percentage in representative figures.

### Statistical analysis

GraphPad Prism 6.0 software (GraphPad Software Inc. San Diego, CA) was used to perform the statistical analysis. Data are given as mean ± standard error of the mean (SEM), or as absolute cell numbers calculated in percent. A Student’s *t*-test with Welch correction and one-way analysis of variance (ANOVA) with a Dunn post-hoc test were used for comparison among different groups. In order to deal post-hoc on non-parametric data, the Mann-Whitney-*U* test was applied, and the Bonferroni adjustment of the p-value to correct for multiple comparisons was performed. A *p* value below 0.05 was considered statistically significant.

## Results

### Study population

A cohort of 18 patients with major trauma (TP) admitted to the ED was enrolled in this study. The majority of the study subjects was male (77.78%) with a mean of 52.21 ± 5.04 years of age. All patients were substantially injured (ISS: 25.69 ± 2.61). A subgroup analysis after frequency-matching of patients according to the ISS (± 6 points) and age showed that patients who developed pneumonia were statistically comparably injured with patients who did not develop clinical complications (ISS: 27.17 ± 3.65 *vs*. 24.43 ± 3.91, Table 1). The injury pattern from trauma was comparable between *no inf.* and *inf*. group (Table 1). The mean stay in the ICU was 13.07 ± 2.95 days and overall time in hospital was 24.80 ± 2.32 days. Comparing mean ICU and hospital stay in patients who did not develop pneumonia with patients developing pneumonia showed significantly prolonged ICU as well as in-hospital stay in patients with infectious complications (ICU: 6.14 ± 2.33 *vs*. 20.00 ± 4.04; hospital stay: 20.25 ± 2.08 *vs*. 30.00 ± 3.55, both *p* < 0.05, Table 1).

**Table 1:** Summary of patient characteristics

Patient characteristics	all *n* = 18	no inf. *n* = 9	inf. *n* = 9	no inf. *vs*. inf. *p* < 0.05
Age (years), mean ± sem	52.21 ± 5.04	48.72 ± 9.18	55.71 ± 4.67	no
Sex (male, %)	14 (77.78%)	7 (77.78%)	7 (77.78%)	no
ISS	25.69 ± 2.61	24.43 ± 3.91	27.17 ± 3.65	no
AIS ≥3 (n, %)				
Head	11 (61.11%)	6 (66.67%)	5 (55.55%)	no
Chest	10 (55.55%)	4 (44.44%)	6 (66.67%)	no
Abdomen	2 (1.11%)	1 (1.11%)	1 (1.11%)	no
Extremity	6 (33.33%)	3 (33.33%)	3 (33.33%)	no
ICU stay (days)	13.07 ± 2.95	6.14 ± 2.33	20.00 ± 4.04	yes
Hospital stay (days)	24.80 ± 2.32	20.25 ± 2.08	30.00 ± 3.55	yes

*AIS*, Abbreviated Injury Scale, *ICU* Intensive Care Unit, *inf.* trauma patients with pneumonia, *ISS* Injury Severity Score, *no inf.* trauma patients without pneumonia; data are presented as mean ± sem unless stated otherwise

### Migratory capacity of isolated neutrophils

**Fig. 1:**
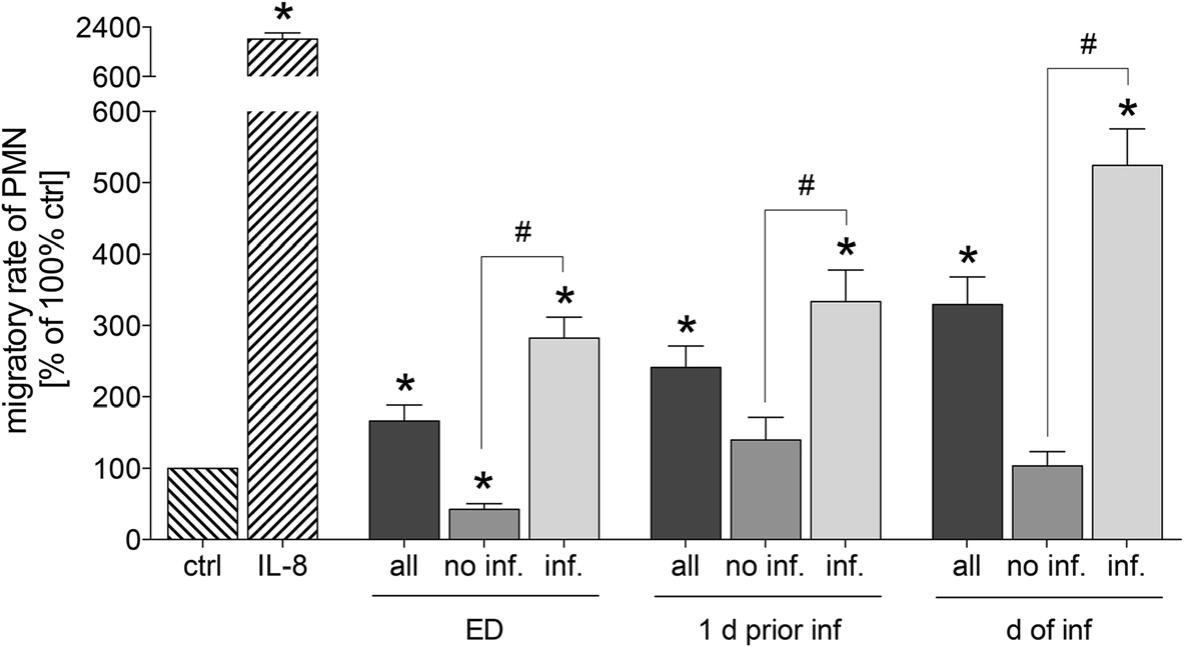
Migratory capacity of neutrophils towards serum samples from major trauma patients (TP). Serum samples were obtained from TP (*n* = 18, all). TP were grouped to *no inf.* without pneumonia (*n* = 9) or *inf*. group with pneumonia (*n* = 9). Samples were obtained at admission to emergency department (ED), a day prior pneumonia diagnosis (*1 d prior inf*) or at the day of diagnosis (*1 d prior inf*). Samples from the equal post-injury days in the corresponding *no inf*. group were used. Neutrophils were isolated from healthy volunteers (HV, *n* = 9). Migratory capacity of neutrophils towards TP’s serum (20% in culture medium) or interleukin (IL)-8 relative to the control (ctrl, 100%) is shown. Data are represented as mean ± SEM. *p* < 0.05 *: *vs.* ctrl, #: *no inf. vs*. corresponding *inf*. group

The incubation of isolated neutrophils with sera from TP showed that the migration rate is significantly higher than the migration rate of untreated control cells (*p* < 0.05, Fig. 1). During the consecutive measurements with samples obtained from ED, day prior infection or at the day of infection (or the corresponding day in the group of patients without pneumonia), this significant increase was observed and continuously rose compared with the control group (ED: 166.5 ± 22.04%, one day prior infection: 241.1 ± 30.08%, and day of infection: 329.5 ± 38.83% *vs*. 100%, respectively; *p* < 0.05, Fig. 1). Stratifying TP into the group with pneumonia development *vs*. no pneumonia showed that the group of patients who did not develop pneumonia had initially lower migratory capacity (ED, no inf.: 42.57 ± 7.72%) compared to the ctrl (*p* < 0.05, Fig. 1). A continuous recovery to baseline was observed in subsequent samples. Incubating isolated neutrophils with sera from TP who developed pneumonia showed significantly increased migratory capacity of these cells over the time course (ED: 282.4 ± 29.52%, one day prior infection: 333.6 ± 44.27%, and day of infection: 524.2 ± 51.42%) as compared to both, ctrl as well as to the corresponding group of TP without pneumonia (*p* < 0.05, Fig. 1).

### Surface expression of CD11b/CD18

**Fig. 2:**
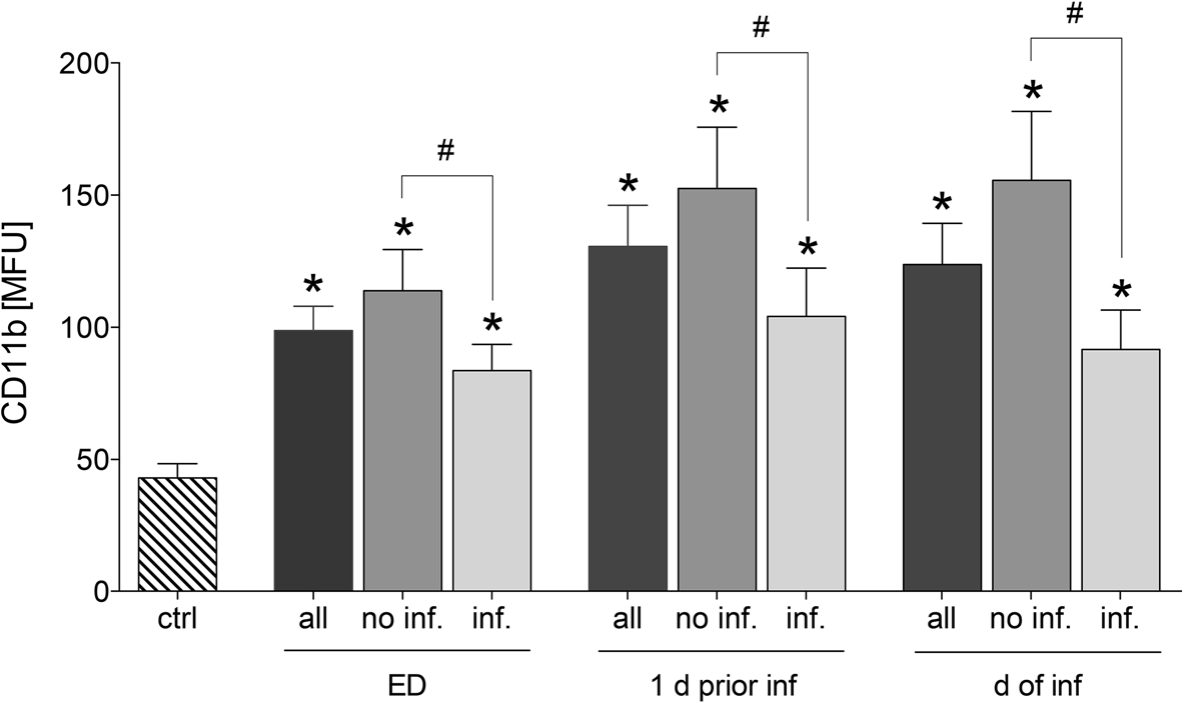
CD11b expression on neutrophils after their incubation with serum samples from major trauma patients (TP). Serum samples were obtained from TP (*n* = 18, all). TP were grouped to *no inf.* without pneumonia (*n* = 9) or *inf*. group with pneumonia (*n* = 9). Samples were obtained at admission to emergency department (ED), a day prior pneumonia diagnosis (*1 d prior inf*) or at the day of diagnosis (*1 d prior inf*). Samples from the equal post-injury days in the corresponding *no inf*. group were used. Neutrophils were isolated from healthy volunteers (HV, *n* = 9). CD11b expression on neutrophils after their incubation with TP’s serum (20% in culture medium) or control without serum addition (ctrl) for two hours was determined by flow cytometry. Data are represented as mean ± SEM. *p* < 0.05 *: *vs.* ctrl, #: *no inf. vs*. corresponding *inf*. group

During the study period the surface expression of CD11b on isolated neutrophils after their incubation with sera from TP remained significantly increased compared to untreated ctrl (ED: 98.70 ± 9.36 MFU, one day prior infection: 130.5 ± 15.58 MFU, and day of infection: 123.7 ± 15.63 MFU *vs*. 42.98 ± 5.41 MFU, *p* < 0.05, Fig. 2). After the incubation of isolated neutrophils with sera from TP either with or without pneumonia, CD11b expression on neutrophils was significantly increased in both groups of TP compared to unstimulated ctrl (*p* < 0.05, Fig. 2). Incubating isolated neutrophils with sera from TP who developed pneumonia showed significantly decreased CD11b expression over the time course compared to the corresponding group of TP without pneumonia (ED: 83.46 ± 9.99 MFU *vs*. 113.9 ± 15.41 MFU, one day prior infection: 104.1 ± 18.4 MFU *vs*. 152.6 ± 23.29 MFU, and day of infection: 91.64 ± 14.94 MFU *vs*. 155.7 ± 26.14 MFU, all *p* < 0.05, Fig. 2).

### Surface expression of CD62L

**Fig. 3:**
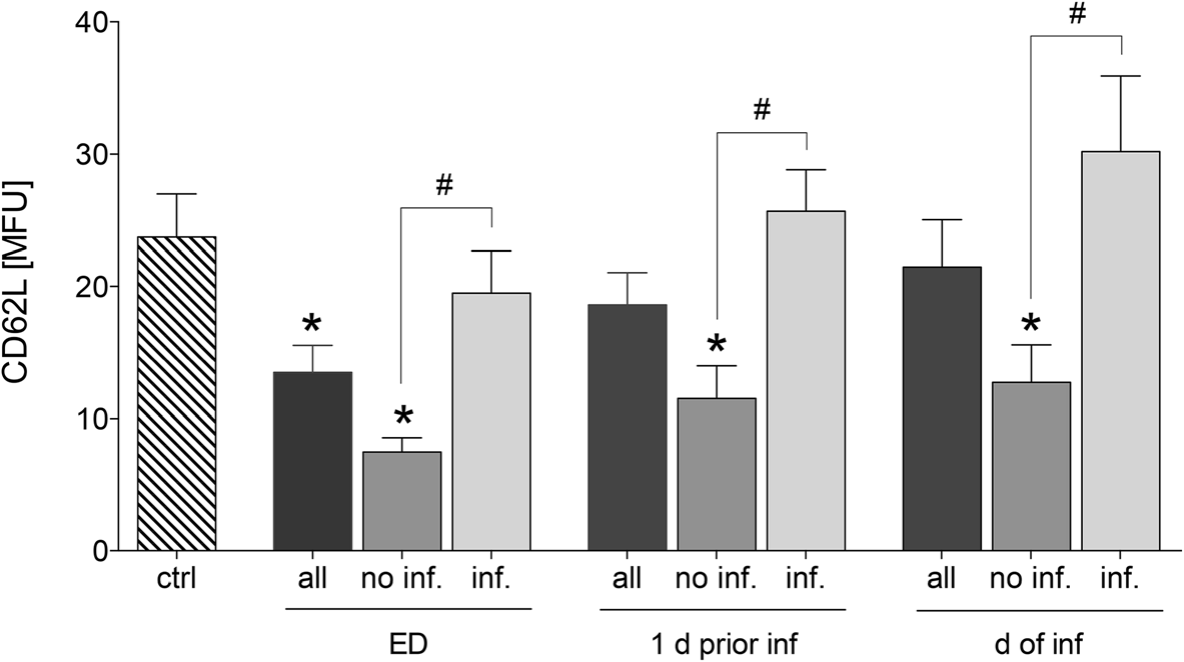
CD62L expression on neutrophils after their incubation with serum samples from major trauma patients (TP). Serum samples were obtained from TP (*n* = 18, all). TP were grouped to *no inf.* without pneumonia (*n* = 9) or *inf*. group with pneumonia (*n* = 9). Samples were obtained at admission to emergency department (ED), a day prior pneumonia diagnosis (*1 d prior inf*) or at the day of diagnosis (*1 d prior inf*). Samples from the equal post-injury days in the corresponding *no inf*. group were used. Neutrophils were isolated from healthy volunteers (HV, *n* = 9). CD62L expression on neutrophils after their incubation with TP’s serum (20% in culture medium) or control without serum addition (ctrl) for two hours was determined by flow cytometry. Data are represented as mean ± SEM. *p* < 0.05 *: *vs.* ctrl, #: *no inf. vs*. corresponding *inf*. group

The CD62L expression on isolated neutrophils after their incubation with sera from TP obtained at ED was significantly decreased compared to untreated ctrl (ED: 13.50 ± 2.04 MFU *vs*. 23.73 ± 3.26 MFU, *p* < 0.05, Fig. 3). This early decrease in CD62L expression recovered nearly to baseline when isolated neutrophils were incubated with sera from patients obtained one day prior infection or at the day of pneumonia diagnosis or the corresponding samples from TP without pneumonia (Fig. 3). After the incubation of isolated neutrophils with sera from TP without pneumonia, CD62L expression on neutrophils was significantly decreased at each study period compared to the corresponding samples from TP with pneumonia or ctrl (ED: 7.49 ± 1.07 MFU *vs*. 19.50 ± 3.19 MFU, one day prior infection: 11.52 ± 2.47 MFU *vs*. 25.67 ± 3.16 MFU, and day of infection: 12.74 ± 2.83 MFU *vs*. 30.19 ± 5.70 MFU, respectively, and ctrl: 23.73 ± 3.26 MFU, all *p* < 0.05, Fig. 3).

### Oxidative burst in isolated neutrophils

**Fig. 4:**
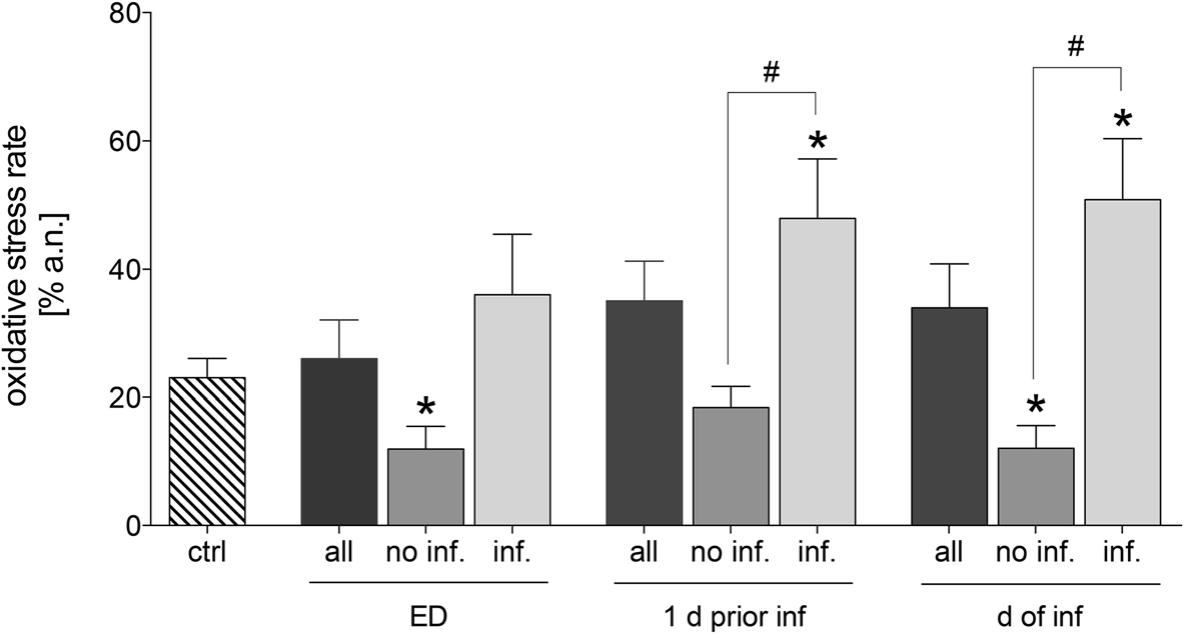
Oxidative burst activity of neutrophils after their incubation with serum samples from major trauma patients (TP). Serum samples were obtained from TP (*n* = 18, all). TP were grouped to *no inf.* without pneumonia (*n* = 9) or *inf*. group with pneumonia (*n* = 9). Samples were obtained at admission to emergency department (ED), a day prior pneumonia diagnosis (*1 d prior inf*) or at the day of diagnosis (*1 d prior inf*). Samples from the equal post-injury days in the corresponding *no inf*. group were used. Neutrophils were isolated from healthy volunteers (HV, *n* = 9). Oxidative burst activity of neutrophils after their incubation with TP’s serum (20% in culture medium) or control without serum addition (ctrl) for two hours was determined by flow cytometry. Data are represented as mean ± SEM. *p* < 0.05 *: *vs.* ctrl, #: *no inf. vs*. corresponding *inf*. group

The incubation of isolated neutrophils with sera from TP showed that the oxidative burst of neutrophils was not markedly altered compared to untreated ctrl cells (Fig. 4). There is a trend to a slightly enhanced oxidative burst activity of neutrophils when incubated with later obtained samples from TP, however this was not statistically significant (Fig. 4). Determination of the oxidative burst activity after incubating neutrophils with samples obtained from TP without pneumonia at ED, showed a significantly reduced oxidative burst activity of neutrophils compared to untreated ctrl (11.96 ± 3.56% *vs*. 23.05 ± 3.01%, *p* < 0.05, Fig. 4). Incubating neutrophils with sera obtained from the same patients one day prior diagnosis of pneumonia showed a slightly lowered oxidative burst, however this was not significant compared to ctrl (Fig. 4). Incubating isolated neutrophils with the sera from TP without infectious complication at the day of pneumonia diagnosis in the corresponding group showed significantly reduced oxidative burst compared to ctrl (12.09 ± 3.53% *vs*. 23.05 ± 3.01%, *p* < 0.05, Fig. 4). The oxidative burst activity trended to an increase when neutrophils were incubated with sera from TP obtained at ED who developed pneumonia (36.03 ± 9.36%), however, this was not significant compared to the ctrl (Fig. 4). During the later time course oxidative burst was significantly increased in neutrophils after their incubation with sera from TP with pneumonia obtained one day prior diagnosis (47.86 ± 9.33%) or at the day of pneumonia diagnosis (50.83 ± 9.54%) as compared to the ctrl or to the corresponding TP group (*p* < 0.05, Fig. 4).

## Discussion

As critical players in the biological host-defense response to trauma, neutrophils are believed not only to protect against microbicidal infections, but also appear to be involved in the pathogenesis of infectious post-injury complications. Enhanced systemic concentrations of proinflammatory cytokines, DAMPs and PAMPs after trauma increase the activity of neutrophils. Once activated, they migrate across damaged endothelium and infiltrate bystander organs [25]. Furthermore, their defensive strategies including oxidative burst activity with ROS production or DNA-trapping become activated [8, 25]. Their role in SIRS occurring early after trauma is out of question. Despite several studies discussed below, the association of their impaired activity in the later post-injury phase with secondary infectious complications such as pneumonia, sepsis or MOF remains to be elucidated [25].

In this study, we have demonstrated that stimulating isolated neutrophils from healthy volunteers with sera samples from trauma patients increased the migratory rate of neutrophils immediately after trauma as well as in the later post-injury course. Interestingly, this increase was caused by neutrophil stimulation with sera samples from trauma patients who developed pneumonia in their clinical course, because here, the migratory rate was higher compared with using samples from patients who did not have infectious clinical post-injury complications. Especially the increased expression of CD11b in all groups accompanied these effects. However, CD62L expression delivered different data, showing that immediately after trauma there was a decrease in CD62L expression. Diminished CD62L expression on circulating neutrophils provides further evidence of activation of blood neutrophils as in vitro stimulation of neutrophils results in shedding of the L-selectin receptor from the cell surface. Stratifying the group of patients with pneumonia showed significantly increased CD11b and significantly decreased CD62L expression of neutrophils after their stimulation with sera from patients without pneumonia compared to patients with pneumonia. These differences persisted during the complete observational period. Summarized, these results suggest that the ratio of CD11b to CD62L and especially increased CD62L expression may reflect the enhanced migratory capacity of neutrophils. Interestingly, ROS production was not markedly altered in general, while it was strongly reduced by applying sera from patients without infectious complications to isolated neutrophils compared to its increase by using sera from patients who developed pneumonia. This data indicate at the high relevance of studying neutrophil function after trauma and their already immediately after trauma modified activity depending on the post-injury clinical course of patients that may have predictive or even therapeutic potential.

Despite numerous studies much of disagreement about the functional role of neutrophils for the development of post-injury complications is found. This is presumably due to the important but dual role of neutrophils with regard to the removal of bactericidal intruders. Following trauma neutrophils are immediately primed for increased migration capacity in response to IL-8 [18]. Furthermore, the authors have observed that neutrophils in the peripheral blood of trauma patients exhibit this increased migration capacity, which is a sign of a primed phenotype, for 3 days after trauma. Thereafter, migration rates declined to baseline levels [18]. In parallel to this data, in our study, enhanced migration rates immediately after trauma were observed as well (Fig. 1). However, this increase persisted during the observational period. Stratifying the patients to those with and without post-injury pneumonia occurrence uncovered that the increased migratory capacity of neutrophils was determined by applying the sera samples from patients who developed pneumonia. Using serum samples from patients who did not develop pneumonia induced initially lower migration rates with a recovery to the baseline in the time course, whereas the use of samples from patients with complication increased continuously migration rates (Fig. 1). Therefore, the data indicate that the blood neutrophil migration rates may be measured as an additional readout to determine the susceptibility of trauma patients for pneumonia and this measurement may also be helpful for the prognosis of trauma patients. Previously, it has been reported that isolated neutrophils from intensive-care patients who developed post-traumatic infections showed a reduced migratory capacity compared to patients without infections [26]. Additionally, recent data underline altered neutrophil migration phenotype in patients with major burns with or without sepsis development [27]. Moreover, reduced neutrophil migration has been shown to be an highly sensitive predictive marker for infections [28]. The mechanisms behind these findings was uncovered by the Hauser group demonstrating that trauma suppresses high-affinity receptors on neutrophils which are important for neutrophil recruitment and activation. The consequence of transiently desensitized receptors on neutrophils after trauma is displayed by suppressed chemotaxis in *ex vivo* and in vitro experiments [29]. Based on these findings the authors suggested that these mechanisms predispose to pneumonia after trauma or other inflammatory conditions. We gained oppose results, however, the neutrophils used in our study were isolated from healthy volunteers and did not undergo a priming situation as described above. Combining our data of strong activating potential of sera from trauma patents with pneumonia with the data from others reporting „desensitation“of neutrophils after trauma, one may hypothesize that there is a profoundly suppressed neutrophil chemosensitation in trauma patients who develop pneumonia. Moreover, our data indicate that factors, which are present immediately after trauma and not at the onset of infection causing the neutrophil „desensitation“in trauma patients, remain to be identified in further studies. Despite this effect, comparable to findings from Bhatia et al. our data indicate that the increased migratory capacity of neutrophils into the lungs increasing their local numbers, as found in ARDS also, may be an important step in the development of post-injury pneumonia.

The migration of neutrophils underlies tightly controlled mechanisms *via* their surface adhesion molecules and the corresponding ligands on endothelial cells. Following trauma, CD11b becomes up-regulated in peripheral neutrophils [14, 17]. Comparable data was found in our study (Fig. 2). Contrasting data have been delivered by others reporting initially unchanged and on day 3 following major trauma down-regulated CD11b expression [18]. In trauma patients who developed sepsis CD11b expression on blood neutrophils was significantly elevated before the patient was diagnosed with sepsis [30]. Interestingly, we found increased CD11b expression after their stimulation with sera from trauma patients either with or without pneumonia. Recently, Hazeldine et al. [12] suggested that combining the data for increased CD11b and reduced L-selectin expression suggests a systemic activation of the circulating neutrophils after trauma [12]. Based on our findings, this approach appears reasonable. Previously, L-selectin expression on circulating neutrophils was reduced after trauma [14, 15]. In line with these findings, CD62L was reduced early after trauma followed by a recovery to baseline in the later course. Interestingly, CD62L expression on neutrophils that were incubated with sera from patients who did not develop pneumonia persisted lowered during the complete study period. However, CD62L expression was increased in neutrophils that were incubated with sera from patients who developed pneumonia (Fig. 3). Recalling the approach by Hazeldine et al. our data support their hypothesis. Yet, we believe that combining the data for increased Mac-1 and reduced L-selectin expression with migration capacity not only suggests a systemic activation of neutrophils after trauma, but also based on differential expression profiles, stratifies patients who are at risk for the development of post-injury pneumonia from those who will not develop pneumonia.

Next to an increased number of activated neutrophils in lungs, pulmonary complications are also characterized by an increased respiratory burst activity of neutrophils [12]. In blunt trauma patients oxidative burst increased time-dependently following n-formyl-L-methionyl-L-leucyl-L-phenylalanine stimulation [31]. We have observed a general trend to an increased oxidative burst activity after trauma, however this was not statistically significant (Fig. 4). One has to keep in mind that here, neutrophils were isolated from healthy volunteers but also that here, the group size is limited to nine per group, and therefore, our study is slightly underpowered to test this specific effect adequately. Nonetheless, our data indicate that there is an increased oxidative burst activity in isolated neutrophils when they are incubated with sera from trauma patients who developed pneumonia compared to the group without pneumonia (Fig. 4). Similar to our data, Lumsdaine et al. reported that neutrophil oxidative burst capacity was found to increase with post-operative complications pneumonia and sepsis [13]. Moreover, the authors postulated that neutrophil oxidative burst capacity was potentially predictive of post-operative development of infective complications following surgery.

As neutrophil oxidative burst capacity is a measure of the host’s immune response and defensive capacity, our data as well as data from others indicate that neutrophils from patients who are at risk for the development of post-injury complications may become early activated following trauma. Differential neutrophil migratory potential as well as altered expression CD11b and CD62L after their stimulation with sera from patients with or without post-injury complications underline this hypothesis. Interestingly, although there are numerous reports about neutrophil function after trauma, human studies about the predictive potential of their altered biology for the outcome after trauma are sparse. Therefore, and based on current findings, it appears convincing that the understanding of the neutrophil biology after trauma will help in finding possible functional targeting strategies to control inflammation in patients who are susceptible to infections.

One another of the key unanswered questions at the moment outlines the above discussed issues. Meanwhile it is clear that the underlying mechanisms to the post-traumatic immunosuppression and the physiological response to sterile and non-sterile trauma go far beyond the SIRS-CARS paradigm [32]. Based on current research data it is difficult to draw conclusions about the causative origin for the immunosuppression after trauma, whether it is caused by either trauma (perforating injury?) itself or even post-traumatic surgery in the later clinical course as a possible DAMP but also PAMP source. In general, trauma mechanism may cause differences in the immune response. Nonetheless, in the present study, there were neither significant differences in trauma mechanism nor specific (diagnosed) e.g., perforating injuries that may represent the PAMP-response. Therefore, our data allow only speculative approaches to address the DAMP/PAMP response. As for example, there is a trend to more chest injuries in the infection group, though this is not significant, it may be associated with increased pulmonary PAMP-translocation and enhanced neutrophil activation. This approach remains to be elucidated in further studies. Looking at the patients’ records has shown that the cohort of included patients suffered from blunt trauma, here. If not considering immediate surgeries after admission (3 patients without and 4 patients with pneumonia), pneumonia developed in 4 patients before surgery and in 4 patients after surgery. One patient without surgery developed pneumonia. On the other hand, all patients except one underwent surgeries as well, and did not develop complications in the comparative group. Additionally, eventual trauma-accompanied infections (e.g., perforating injury) that may have altered the immune status can be excluded. Therefore, there is no evident indication, neither for a possible trauma-accompanied infection source, nor for the surgery as the second-hit causing infectious complications. One has to consider that the samples which were obtained at ED were acquired before surgeries were performed. Interestingly, the differences in the activation of neutrophils between the groups were given immediately after admission. On the other hand, the ISS was slightly higher in the group of patients who developed pneumonia in their later post-injury course speaking for eventually larger surgeries. However, apparently there must be some other influencing factors early after trauma which were not considered here. Due to these early differences, there are also inherent differences between the immune systems of those who did and did not develop pneumonia. These early changes may be associated with the pneumonia development in the later post-injury course. This issue cannot be excluded by the present study, and in general, this issue can be addressed only by further larger clinical-experimental studies. Therefore, the data on the influence of sterile versus non-sterile trauma are limited, and the presented manuscript underlines further the importance and the relevance of measuring a patients immune status immediately after trauma and before treatment in order to 1.) finding the ideal clinical approach and 2.) to understanding the differences and influence of sterile versus non-sterile trauma on the immune status.

Nevertheless, a limitation of this study is the relatively small sample size making appropriate statistical analysis difficult. Although, there were relevant and novel trends observed regarding our data, possibly, with a larger sample size, these differences may become more convincing.

## Conclusions

This study investigated differential biology of isolated neutrophils from healthy volunteers upon their incubation with sera obtained from trauma patients, who either did or did not suffer from pneumonia in their clinical post-injury course. Our findings indicate that applying sera from patients at risk for pneumonia development differentially and early activate healthy neutrophils compared to the use of sera from patients who are not at risk for post-injury complication. Studies about the differential neutrophil biology and their immediately after trauma modified activity depending on the post-injury clinical course are warranted, and may deliver possible functional targeting strategies to control inflammation in patients who are susceptible to infections.

## Abbreviations

CARS: Compensatory anti-inflammatory response syndrome
CD: Cluster of differentiation
CMV: Cytomegalovirus
Ctrl: Control
DAMP: Damage-associated molecular pattern
ED: Emergency department
HEPES: 4-(2-hydroxyethyl)-1-piperazineethanesulfonic acid
HIV: Human immunodeficiency virus
ICU: Intensive care unit
IL: Interleukin
Mac-1: Macrophage-1 antigen
MARS: Mixed antagonist response syndrome
MFU: Mean fluorescence unit
MODS: Multi organ dysfunction syndrome
MOF: Multiple organ failure
PAMP: Pathogen-associated molecular pattern
PE: Phycoerythrin
PMN: Polymorphonuclear leukocytes
ROS: Reactive oxygen species
RPMI: Roswell Park Memorial Institute
SIRS: Systemic inflammatory response syndrome
TP: Trauma patients
